# Gefährdungsbeurteilung psychischer Belastung: Geeignete Analyseinstrumente für das Sozial- und Gesundheitswesen

**DOI:** 10.1007/s40664-021-00450-w

**Published:** 2021-11-26

**Authors:** Maren Kersten, Agnessa Kozak, Mareike Adler, Claudia Wohlert, Susanne Stamer, Sabine Gregersen

**Affiliations:** 1 Abteilung Arbeitsmedizin, Gefahrstoffe und Gesundheitswissenschaften, Berufsgenossenschaft für Gesundheitsdienst und Wohlfahrtspflege (BGW), Pappelallee 33/35/37, 22089 Hamburg, Deutschland; 2 Institut für Versorgungsforschung in der Dermatologie und bei Pflegeberufen (IVDP), Competenzzentrum für Epidemiologie und Versorgungsforschung bei Pflegeberufen (CVcare), Universitätsklinikum Hamburg-Eppendorf, Hamburg, Deutschland

**Keywords:** Personalbefragung, Beobachtungsverfahren, Gruppendiskussion, Kriterienkatalog, Auswahl, Personel survey, Observation procedures, Group discussion, Kriterienkatalog, Selection

## Abstract

**Hintergrund:**

Die Gefährdungsbeurteilung psychischer Belastung hat zum Ziel, die durch die Arbeit verbundenen Gefährdungen zu beurteilen, um Maßnahmen zur gesundheitsgerechten Gestaltung der Arbeit abzuleiten. Allerdings ist das Angebot an Verfahren für die Ermittlung der psychischen Belastung umfangreich und unübersichtlich. Vor diesem Hintergrund wird für das Sozial- und Gesundheitswesen ein reduzierter und strukturierter Überblick an Instrumenten vorgestellt.

**Methode:**

Für die Identifizierung geeigneter Instrumente wurde eine umfangreiche Suche durchgeführt. Vor Recherchebeginn wurden Kriterien definiert, um die identifizierten Instrumente auf Eignung zu prüfen. Zum einen gab es Mindestanforderungen, die erfüllt sein mussten, damit das Instrument in den Review-Prozess aufgenommen wurde, und zum anderen Strukturierungskriterien, die sich in beschreibende und bewertende Aspekte unterteilten.

**Ergebnisse:**

Die Recherche identifizierte insgesamt 83 Instrumente für die Gefährdungsbeurteilung psychischer Belastung (GBU Psyche); nach der ersten Sichtung wurden 58 von diesen zur weiteren Eignung im Review-Prozess übernommen. Abgeschlossen wurde das Gesamtreview bisher für 44 Verfahren aus der unsystematischen Suche. Davon wurden 19 Verfahren als geeignet eingestuft und in einer Übersichtsmatrix strukturiert dargestellt. Das Review für die 14 Verfahren aus der systematischen Recherche erfolgt voraussichtlich bis Mitte 2022 und ist Teil eines kontinuierlichen Review-Prozesses.

**Diskussion:**

Die Vielzahl an identifizierten Verfahren für die GBU Psyche (Gefährdungsbeurteilung psychischer Belastung) zeigt deutlich die Sinnhaftigkeit und Relevanz, eine begrenzte, praxiserprobte sowie qualitätsgesicherte Auswahl an Instrumenten zu treffen. Die ebenfalls in diesem Artikel dargestellten Kriterien zur Bewertung der Instrumente, machen die getroffene Auswahl transparent.

## Abstract (en)

**Background:**

The aim of the risk assessment for mental stress is to evaluate the hazards associated with the work in order to derive measures for the healthy design of the work. The range of procedures for assessment of mental stress is, however, extensive and unclear. Against this background, this article presents a reduced and structured overview of instruments for social and healthcare services.

**Methods:**

A comprehensive search was carried out to identify appropriate tools. Prior to beginning the search, criteria were defined to test the suitability of the identified tools. For one thing, there are minimum requirements that must be fulfilled so that the tool is accepted for the review process. There are also structuring criteria that can be divided into descriptive and evaluative aspects.

**Results:**

The search identified a total of 83 instruments for risk assessment of mental stress (GBU Psyche), of which 58 were accepted for a further suitability review process following initial evaluation. The comprehensive review of 44 services from the nonsystematic search has so far been completed. Of these, 19 procedures were deemed suitable and are presented in a structured overview matrix. The review of the 14 procedures from the systematic search is projected to be finished by mid-2022 and is part of the continual review process.

**Discussion:**

The numerous procedures identified for risk assessment of mental stress clearly show that it is reasonable and relevant to find a limited selection of tools that have been tested in practice and are quality assured. The criteria for evaluating the tools, which are also presented in this article, render this selection transparent.

Arbeitgeber*innen sind laut Arbeitsschutzgesetz verpflichtet, eine Gefährdungsbeurteilung durchzuführen, um menschengerechte Arbeitsbedingungen zu gewährleisten. Psychische Gefährdungen können sich aus der Arbeitsaufgabe, der Arbeitsorganisation, den sozialen Beziehungen und der Arbeitsumgebung ergeben. Zum Schutz der Gesundheit sollten zielgerichtete und wirkungsvolle Arbeitsschutzmaßnahmen abgeleitet werden. Dafür wurden zunächst psychische Gefährdungen für die betreffenden Tätigkeiten oder Bereiche mithilfe von Analyseinstrumenten umfassend ermittelt. Für die Branche Sozial- und Gesundheitswesen zeigt dieser Beitrag eine Auswahl geeigneter Instrumente auf.

## Hintergrund

Angesichts der Veränderung in der Arbeitswelt (z. B. Restrukturierungsmaßnahmen, Flexibilitäts- und Mobilitätsanforderungen an Beschäftigte) kommt der psychischen Belastung eine besondere Bedeutung zu [7]. Bei der psychischen Belastung handelt es sich um „die Gesamtheit der erfassbaren Einflüsse, die von außen auf den Menschen zukommen und psychisch auf ihn einwirken“ [10]. Der Zusammenhang zwischen Arbeitsbedingungen einerseits und Gesundheit andererseits wird bereits intensiv erforscht [28]. Eine ungünstige psychische Belastung bei der Arbeit kann zur Entwicklung von psychischen Erkrankungen, z. B. Depression und Angststörung, beitragen [23] und darüber hinaus an der Entstehung vieler körperlicher Erkrankungen, z. B. Herz-Kreislauf- oder Muskel-Skelett-Erkrankungen, beteiligt sein [18, 23].

## Durchführung der Gefährdungsbeurteilung Psyche

Das Arbeitsschutzgesetz verpflichtet Arbeitgeber*innen dazu, auf der Basis einer Beurteilung der Arbeitsbedingungen erforderliche Maßnahmen des Arbeitsschutzes festzustellen, umzusetzen und im Hinblick auf ihre Wirksamkeit zu kontrollieren. Bei dieser Gefährdungsbeurteilung sind auch psychische Belastungen der Arbeit zu berücksichtigen [6]. Die GBU Psyche hat zum Ziel, die durch die Arbeit verbundenen Gefährdungen zu beurteilen, um Maßnahmen zur gesundheitsgerechten Gestaltung der Arbeit abzuleiten. Sie leistet damit einen entscheidenden Beitrag zur menschengerechten Gestaltung der Arbeit. Das grundsätzliche Vorgehen bei der GBU Psyche gleicht dem Prozess bei anderen Gefährdungen, wie z. B. Lärm, jedoch werden andere Instrumente zur Messung verwendet. Die sieben Prozess-Schritte der Gefährdungsbeurteilung, die absolviert werden müssen, lauten: Arbeitsbereiche und Tätigkeiten festlegen, Gefährdungen ermitteln, Gefährdungen beurteilen, Maßnahmen festlegen, Maßnahmen durchführen, Wirksamkeit prüfen und Gefährdungsbeurteilung fortschreiben.

Trotz der gesetzlichen Forderung und der zunehmenden Bedeutung der psychischen Belastung gibt es Hinweise, dass nur eine Minderheit der Unternehmen die GBU Psyche umsetzt [4, 21]. Schätzungen zufolge erfassen lediglich 30 % der Unternehmen die psychische Belastung in der Gefährdungsbeurteilung [12].

Eine repräsentative Befragung in Deutschland ergab, dass eine der zentralen Hürden bei der Erfassung der psychischen Belastung liegt, gerade bei kleineren Einrichtungen. Bei den Verantwortlichen in den Einrichtungen bestehen Unsicherheiten bezogen auf die konkreten Inhalte sowie die Art und Weise der Erfassung der Gefährdungen [3]. Eine zentrale Herausforderung stellt die Passung zwischen dem Instrument und den tätigkeitsspezifischen Anforderungen dar. Deshalb ist eine branchenspezifische Sichtung von geeigneten Instrumenten zur Erfassung eine wichtige Unterstützung der Arbeitgeber*innen bei der Umsetzung der GBU Psyche.

## Bedeutung der Branche Sozial- und Gesundheitswesen

Die Branche Sozial- und Gesundheitswesen hat eine erhebliche ökonomische Bedeutung für den Standort Deutschland [8]. Die Anzahl der Beschäftigten in dieser Branche ist in den vergangenen Jahren deutlich gestiegen [1, 37]. Die Arbeitsbedingungen im Sozial- und Gesundheitswesen haben sich seit einiger Zeit aufgrund von Ökonomisierung (z. B. Optimierung von Patienten-Pflegepersonal-Relation), Umstrukturierungen (z. B. Einführung von Fallpauschalen) und den Auswirkungen des demografischen Wandels verändert. Die Arbeits- und Gesundheitssituation ist gekennzeichnet durch hohe Arbeitsintensität und Arbeit an der Grenze der Leistungsfähigkeit [7, 17]. Darüber hinaus weisen die Arbeitsbedingungen häufig ungünstige Konstellationen aus hohen Stressoren (z. B. Überstunden) und geringen Ressourcen (z. B. geringe zeitliche Handlungsspielräume) auf [7]. Im Sozial- und Gesundheitswesen ist die Arbeit durch die Interaktionen mit Menschen in schwierigen Lebenssituationen oder mit Personen mit angeborenen oder erworbenen Behinderungen, die häufig mit herausforderndem Verhalten einhergehen, geprägt.

Die spezifischen Tätigkeiten sollten sich bei der Auswahl von geeigneten Instrumenten für die GBU Psyche widerspiegeln. Nachfolgende Merkmale sind hierbei u. a. zu berücksichtigen:

- emotionale Inanspruchnahme – auch mit dem Thema Gewalt gegenüber Mitarbeitenden [29, 34],
- überlange Arbeitszeit, z. B. bei Klinikärzt*innen [33],
- quantitative Anforderungen, z. B. hoher Zeitdruck [7, 17, 26],
- qualitative Anforderungen, z. B. Umgang mit dementen Bewohner*innen [35].

Die Auswirkungen der COVID-19-Pandemie im Jahr 2020 und 2021 auf die Tätigkeiten im Sozial- und Gesundheitswesen haben in vielen Bereichen zu einer weiteren Arbeitsverdichtung und zu kritischen Veränderung geführt [15, 32]. Unter Berücksichtigung dieser aktuellen Entwicklungen im Sozial- und Gesundheitswesen ist die GBU Psyche von besonderer Bedeutung [5].

Das umfangreiche und unübersichtliche Angebot an Analyseinstrumenten ist für Unternehmen eine Herausforderung bei der Auswahl eines geeigneten Instruments. Um für das Sozial- und Gesundheitswesen eine praxiserprobte und qualitätsgesicherte Auswahl an Analyseinstrumenten zu identifizieren, hat sich eine Arbeitsgruppe gegründet, bestehend aus Mitarbeiterinnen der Berufsgenossenschaft für Gesundheitsdienst und Wohlfahrtspflege (BGW) und dem Competenzzentrum Epidemiologie und Versorgungsforschung bei Pflegeberufen (CVcare) am Universitätsklinikum Hamburg-Eppendorf (UKE). Anhand von a priori entwickelten, standardisierten Kriterien wurden Analyseinstrumente zur Beurteilung der psychischen Gefährdungen bei der Arbeit in einem Peer-Review-Prozess begutachtet. Das Ziel ist ein qualitätsgesicherter und strukturierter Überblick über branchenspezifische, aber auch branchenunabhängige Instrumente für die betrieblichen Arbeitsschutzakteure und Anwender*innen. In der hier vorgestellten Arbeit handelt es sich um eine Momentaufnahme eines kontinuierlichen Prozesses, bei dem Instrumente bei einer Neuauflage erneut geprüft sowie neu entwickelte Verfahren in den Begutachtungsprozess aufgenommen werden.

## Methode

Bei den gesichteten Analyseverfahren handelt es sich um Personalbefragungen, Beobachtungs- und Gruppendiskussionsverfahren. In einem ersten Schritt wurde zunächst eine unsystematische Literatursichtung durchgeführt. Diese wurde in einem zweiten Schritt durch eine systematische Literaturrecherche ergänzt. Im dritten Schritt haben zwei Reviewer die identifizierten Analyseinstrumente nach vorab festgelegten Kriterien unabhängig bewertet; jeder Reviewer führte die Instrumentenbewertung separat durch. In einem Treffen der zwei Reviewer wurden sämtliche Merkmale konsekutiv abgeglichen; bei Unstimmigkeit haben die beiden Reviewer darüber diskutiert. Für den Fall, dass keine Einigung gelang, wurde die Unstimmigkeit in der gesamten Arbeitsgruppe besprochen und ein Konsens gefunden.

Für den ersten Schritt der unsystematischen Literatursichtung wurden Instrumente aus der Fachliteratur, der BAuA Toolbox [24], Kongressen sowie Angebote der Unfallversicherungsträger und kommerzieller Anbieter einbezogen.

Im zweiten Schritt wurde eine systematische Literaturrecherche durchgeführt, um das Risiko eines Selektionsbias zu reduzieren; die Recherche wurde in den Datenbanken PubPsych und PsycInfo vorgenommen. Bei der Datenbank PubPsych handelt es sich um eine Meta-Datenbank. Der Suchstring setzte sich aus drei Dimensionen zusammen: Instrumente, psychische Belastungen und psychische Beeinträchtigung, für die jeweils Synonyme gebildet wurden (Tab. 1). Die Begriffe in den Spalten wurden jeweils mit „OR“ verknüpft, die Begriffe in den Zeilen jeweils mit „AND“.

**Table 1:** 

**Instrumente**	**AND**	**Psychische Belastung**	**AND**	**Psychische Beeinträchtigung**
Analyse*		psychische* Belastung		Beanspruchung*
Befragung		Arbeitsbedingung*		Wohlbefinden
Beobachtung*		Arbeitscharakteristika		psychische Gesundheit
Gruppendiskussion		*ressource*		Gesundheit
Gefährdungsbeurteilung		*stressor*		
*instrument		*anforderungen		
Fragebogen		*arbeitsmerkmale		
Workshop		psychomental		
Messung		Arbeitsumgebung		
Verfahren		Arbeitsplatzmerkmale		
Arbeitsplatzanalyse		Arbeitsinhalte		
Arbeitsplatzbeurteilung		Arbeitsorganisation		
Beurteilung		neue Arbeitsformen		
Umfrage		soziale Beziehungen		
betrieblich* Gesundheits*				
Organisationsentwicklung				
Arbeitsgestaltung				

Der Veröffentlichungszeitraum der Artikel wurde auf die Jahre 1970 bis 2020 eingegrenzt. Begriffe wie Störung*, Patient*, Krank* und *Therapie wurden mit *NOT-Operatoren* verknüpft, um Literaturtreffer sinnvoll einzugrenzen. Die in der Tab. 1 dargestellten Suchbegriffe wurden für die Abfrage der englischsprachigen Datenbank PsycInfo übersetzt. Gesucht wurde nach Instrumenten in deutschsprachigen Veröffentlichungen, die ab dem Jahr 1970 veröffentlicht wurden (letzte Aktualisierung der Datenbankabfrage am 22.04.2020). Um die Auswahl einzugrenzen, wurden Studien, die psychische Erkrankungen untersucht hatten, von vornherein ausgeschlossen (Limitation: „non disordered populations“).

Zunächst wurden die Titel und Abstracts gesichtet. Anschließend wurden ausgewählte Volltexte dahingehend geprüft, ob sie ein Verfahren in deutscher Sprache zur Durchführung der GBU Psyche beschreiben.

### A-priori-Entwicklung von Kriterien zur Sichtung der Analyseinstrumente

Vor Recherchebeginn wurden Kriterien definiert, um die identifizierten Instrumente auf Eignung zu sichten. Bestandteil dieses Kriterienkatalogs waren zunächst sog. Mindestanforderungen, die erfüllt sein mussten, damit das Instrument in den Review-Prozess aufgenommen wurde. Dabei handelt es sich bspw. um die Kriterien, ob die Instrumente theorie- oder wissenschaftsbasiert entwickelt wurden und ob sie praxisbewährt sind (Tab. 2). Die Mindestanforderungen sind sowohl für die Instrumente der unsystematischen als auch für die der systematischen Recherche geprüft worden.

**Table 2:** 

**Mindestanforderungen**	
*Obligatorisch*	Erhebung psychischer Belastung
	Theoriebasierung
	Personenübergreifende Auswertung
	Praxisbewährtheit
	Zugänglichkeit
**Strukturierungskriterien**	
*Beschreibend*	Branchenspezifisch oder branchenübergreifend
	Überblick oder detaillierte Analyse psychischer Belastung
	Abfrage von Beanspruchung
	Differenzierung zwischen psychischer Belastung und psychischer Beanspruchung
	Instrument liegt in mehreren Sprachen vor
	Möglichkeiten der Onlinenutzung
	Verständlichkeit der Fragen
	Handlungsleitfaden als erklärendes Dokument vorhanden
*Bewertend*	Handlungsleitfaden: – Informationen zur Umsetzung des Verfahrens – Hinweise zur Auswertung der Ergebnisse – Übertragung der Ergebnisse auf Gefährdungsbeurteilung möglich
	Angaben zur Dauer des Ausfüllens/Anwendung und zur Durchführung des Instruments
	Validität
	Kosten des Instruments
	Verfügbarkeit von Referenzwerten, Normwerten etc. (Möglichkeit zur Beurteilung und Einordnung der Gefährdung)
*GDA-Kriterien* *bewertend*	Arbeitsorganisation: insbesondere Arbeitsintensität und Arbeitszeit
	Arbeitsinhalte/-aufgaben: insbesondere Handlungsspielraum
	Soziale Beziehungen: insbesondere zu Vorgesetzten
	Arbeitsumgebung: insbesondere Belastung durch Lärm
*GDA-Kriterien* *nichtbewertend*	Neue Arbeitsformen: räumliche Mobilität, atypische Arbeitsverhältnisse, zeitliche Flexibilisierung, reduzierte Abgrenzung zwischen Arbeit und Privatleben

Waren die Mindestanforderungen erfüllt, haben zwei Reviewer das Instrument unabhängig voneinander anhand von Strukturierungskriterien, inklusive der Merkmale der Gemeinsamen Deutschen Arbeitsschutzstrategie (GDA; [11]; Tab. 2), bewertet. Sofern wichtige weiterführende Informationen zur Bewertung der Kriterien fehlten, wurden die Autoren*innen bzw. Herausgeber*innen der Analyseinstrumente kontaktiert und um Auskunft gebeten.

Die qualitative Validierung der Kriterien erfolgte in verschiedenen Expertengruppen (z. B. mit Wissenschaftler*innen, Berater*innen, Aufsichtspersonen).

Die Strukturierungskriterien unterteilen sich in beschreibende und bewertende Aspekte. Um die Art des Instruments zu beschreiben, wurden Kriterien genutzt, wie z. B. orientierend (<30 Items) vs. vertiefend (ab 30 Items) für Fragebögen sowie branchenspezifisch vs. branchenübergreifend für alle Instrumente. Eine derartige Kategorisierung ist notwendig für eine zu entwickelnde Übersicht über die Art der Verfahren. Bei bewertenden Strukturierungskriterien werden Punkte vergeben, z. B. bei der Erfüllung der GDA-Merkmale. Dieses Vorgehen soll eine begrenzte, praxiserprobte und transparente Auswahl für Anwender*innen ermöglichen. Eine tätigkeitsspezifische Auswahl wurde dadurch erreicht, dass bspw. die GDA-Merkmale speziell für die Anforderungen des Sozial- und Gesundheitswesens gesichtet wurden (z. B. emotionale Inanspruchnahme, Zeitdruck, häufige Unterbrechungen). Instrumente, mit den höchsten Punktzahlen wurden als geeignet in die Übersichts-Matrix aufgenommen (Abb. 3).

Die Überprüfung der Kriterien wurde auf unterschiedlichen Ebenen vorgenommen: Die erste Ebene, die Erarbeitung der Kriterien, bezog auch die Anlage 3 der Empfehlungen zur Umsetzung der Gefährdungsbeurteilung psychischer Belastung der GDA [2] mit ein. Auf der Ebene zwei, nach der Erstellung einer Kriterienliste, wurden erste Instrumentenbegutachtungen durchgeführt und daraufhin die Kriterien verfeinert. Schließlich wurden auf der dritten Ebene Expert*innen in internen und externen Gremien einbezogen (Aufsichtspersonen, Fachkräfte für Arbeitssicherheit, Betriebsärzte, Gesundheits- und Pflegewissenschaftler, Berater und Arbeitspsychologen).

## Ergebnisse

**Figure 1:**
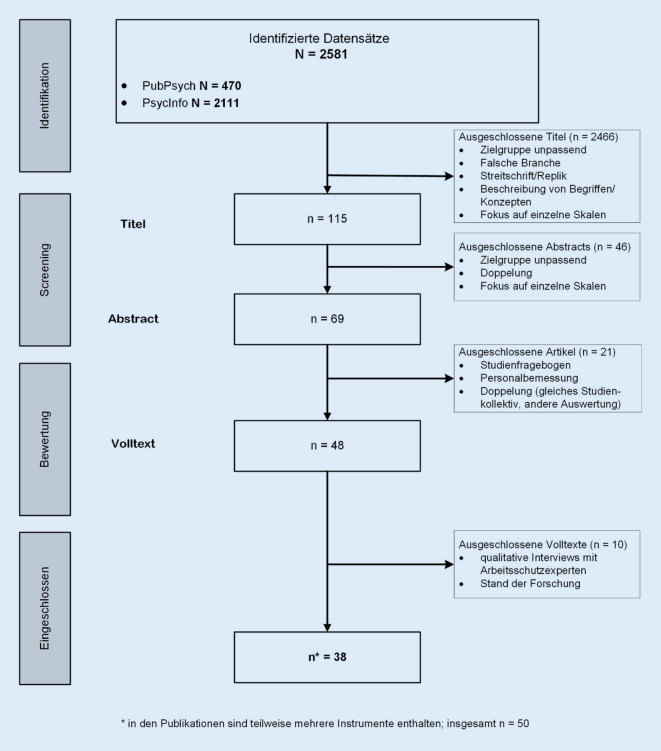

Insgesamt wurden 83 Instrumente durch die Recherchen identifiziert, von denen 58 die Mindestanforderungen erfüllten. Die identifizierten Instrumente entstammten dabei sowohl der systematischen Recherche (Abb. 1) als auch der unsystematischen Suche.

**Figure 2:**
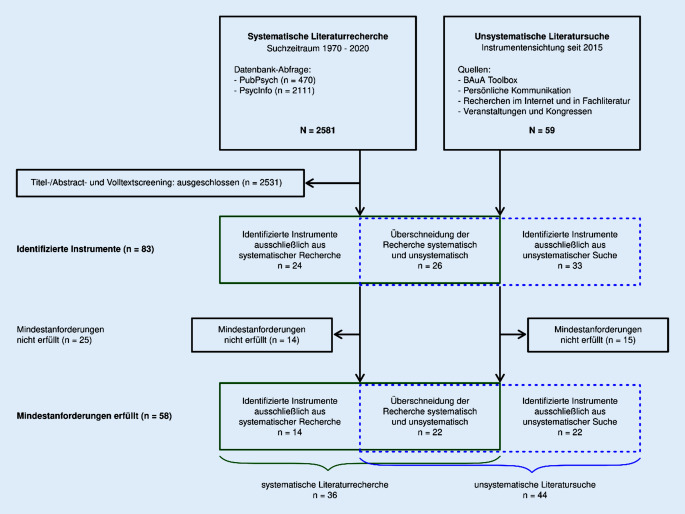

Die eingeschlossenen Artikel der systematischen Recherche ergaben letztlich 50 Instrumente für die GBU Psyche (Abb. 2). Von diesen erfüllten 36 die Mindestanforderungen. In der unsystematischen Suche wurden 59 Instrumente identifiziert, von denen 44 die Mindestanforderungen erfüllten. In der Schnittmenge von systematischer und unsystematischer Suche befanden sich letztlich 22 Instrumente.

**Figure 3:**
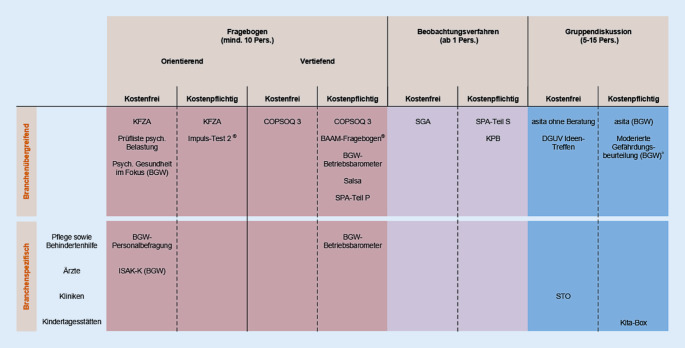

Abgeschlossen wurde das Review bisher für 44 Verfahren aus der unsystematischen Suche, davon wurden 19 Verfahren als geeignet für das Sozial- und Gesundheitswesen identifiziert (Abb. 3). Das Review für die 14 verbleibenden Verfahren aus der systematischen Recherche erfolgt voraussichtlich bis Mitte des Jahres 2022 und ist Teil des kontinuierlichen Review-Prozesses.

Sämtliche identifizierten Instrumente der Literaturrecherche können bei den Autoren angefragt werden.

### Übersicht der geeigneten Instrumente

Für die GBU Psyche gibt es drei verschiedene Verfahrensarten, dabei handelt es sich um Personalbefragungen, Beobachtungs- und Gruppendiskussionsverfahren; für alle drei Arten konnten für das Sozial- und Gesundheitswesen geeignete Instrumente identifiziert werden.

Aus den bewertenden Kriterien (bspw. Handlungsleitfaden vorhanden, ermöglichen die Ergebnisse eine Beurteilung von Gefährdungen z. B. durch Referenzwerte oder qualitative Beurteilung) ergab sich am Ende des Prozesses eine Punktzahl. Bei einem Wert ≥20 Punkte wurde das Instrument in die Matrix aufgenommen. Maximal konnten 31 Punkte erreicht werden.

Einen Überblick der geeigneten Verfahren gibt die Matrix in Abb. 3. Diese strukturiert die Instrumente in Fragebögen, Beobachtungs- sowie Gruppendiskussionsverfahren, wobei Fragebögen noch einmal in orientierend (<30 Items) und vertiefend (ab 30 Items) unterteilt wurden. Eine weitere Strukturierung fand in branchenübergreifende und branchenspezifische sowie in kostenfreie und kostenpflichtige Instrumente statt.

Einige Instrumente sind sowohl in den Kategorien kostenfrei und kostenpflichtig geführt. Der Grund hierfür ist, dass sie kostenfrei zugänglich sind. Sollten externe Dienstleister*innen hinzugezogen werden, so ist die Nutzung kostenpflichtig. Ein weiteres Instrument befindet sich sowohl in der Kategorie branchenspezifisch als auch branchenübergreifend; in diesem Fall hat das Instrument einen modular aufgebauten Fragebogen, der branchenspezifisch, z. B. in der stationären Altenpflege, sowie auch branchenübergreifend, bspw. bei einem großen Träger der ambulanten, stationären Behindertenhilfe und deren Werkstattbereich, eingesetzt werden kann.

Bei der Matrix handelt es sich um die Darstellung eines aktuellen Zwischenstands, da Verfahren regelmäßig von den Autor*innen überarbeitet werden [16, 27], oder es werden neue Instrumente entwickelt [36]. Die verbleibenden 14 Instrumente aus der systematischen Recherche werden im Jahr 2022 den Review-Prozess durchlaufen und ggf. die Matrix erweitern. Die Sichtungsarbeit strukturiert sich somit in mehrere Begutachtungswellen.

Für die bis zu diesem Zeitpunkt als geeignet identifizierten Instrumente wird folgend auf den Review-Prozess eine Kurzbeschreibung, ein sog. Steckbrief angefertigt. Dieser soll den Anwender*innen einen schnellen und detaillierten Einblick in das jeweilige Verfahren ermöglichen.^1^

## Diskussion

Die vorliegende Arbeit identifiziert anhand von Recherchen insgesamt 83 Instrumente für die GBU Psyche. Anhand zuvor definierter Kriterien wurden die einzelnen Instrumente auf ihre Eignung und Einsatzmöglichkeiten geprüft, sodass eine reduzierte Anzahl von Verfahren zur Durchführung der GBU Psyche für das Sozial- und Gesundheitswesen vorgestellt werden kann.

Die Ergebnisse zeigen, dass durch die Vielzahl an identifizierten Verfahren auf dem Anbietermarkt für die GBU Psyche, die Sinnhaftigkeit und Relevanz, eine transparente, begrenzte, praxiserprobte sowie qualitätsgesicherte Auswahl von Instrumenten zu treffen wichtig ist. Arbeitsschutzakteure sowie Anwender*innen können ihren Anforderungen entsprechend (z. B. Branchenspezifik) ein geeignetes Verfahren für ihre Einrichtung auswählen. Ein innerbetrieblicher Diskussions- und Entscheidungsprozess kann mithilfe der Matrix und den darin enthaltenen unterschiedlichen Herangehensweisen initiiert und im weiteren Verlauf die Auswahl eines entsprechenden Instruments zielgerichtet getroffen werden.

Bei der an Kriterien orientierten Sichtung geeigneter Instrumente für die Branche Sozial- und Gesundheitswesen war die Berücksichtigung bspw. von emotionalen Anforderungen aus den Arbeitsinhalten (z. B. Umgang mit Personen mit herausfordernden Verhaltensweisen), der Arbeitszeit (z. B. Dienstplangestaltung) sowie die Vereinbarkeit von Beruf- und Privatleben (z. B. nicht aus dem Frei geholt werden) besonders wichtig. Diese Arbeitsmerkmale haben insbesondere im Sozial- und Gesundheitswesen einen deutlichen Einfluss auf die Gesundheit der Beschäftigten [7, 29, 33].

Die Auswahl eines geeigneten Instruments ist von den betrieblichen Rahmenbedingungen (z. B. Betriebsgröße, Branche, Tätigkeitsbereiche, finanzielle Ressourcen) sowie von der Art der Arbeitsanforderungen abhängig. Im Allgemeinen wird zunächst ein orientierendes Verfahren (z. B. standardisierte schriftliche Personalbefragung) gewählt, um einen Überblick darüber zu erhalten, ob und wo es Belastungsschwerpunkte gibt. Werden Problembereiche identifiziert, sollte ein vertiefendes Verfahren (z. B. moderierter Analyseworkshop) für ausgewählte Bereiche angewendet werden. Aus der Anwendung eines geeigneten Instruments resultiert noch keine Rechtssicherheit, vielmehr kommt es auf die Einhaltung der sieben Prozessschritte an. Unabhängig von der ausgewählten Methode steht der Prozess der GBU im Vordergrund. Die professionelle Berücksichtigung der Prozessschritte gewährleistet die Nachhaltigkeit der Ergebnisse und die Rechtssicherheit. Mit dieser hier genannten Auswahl werden Beispiele geeigneter Instrumente beschrieben. Es können auch andere Verfahren für die Gefährdungsbeurteilung eingesetzt werden.

Seit 2018 prüfen die gesetzlichen Unfallversicherungsträger, ob ihre versicherten Unternehmen die GBU Psyche verantwortungsbewusst durchführen.

Die GBU Psyche wird nach wie vor noch längst nicht in jeder Einrichtung durchgeführt [12]; ein Hindernis dafür kann das fehlende Wissen sein, was psychische Belastung ist, und sich in der uneinheitlichen Sprachverwendung ausdrückt und somit zu Verwirrung führt. Außerdem zeigt sich ein Mangel an Wissen zur Messbarkeit von psychischer Belastung [4]. Durch Informationen wie bspw. eine Auswahl an geeigneten Instrumenten kann dieses Hindernis verringert werden.^2^

Eine Limitation der vorliegenden Arbeit ist es, dass weitere Instrumente existieren, von denen die Arbeitsgruppe keine Kenntnis hat und die ebenfalls geeignet sein können, die GBU Psyche im Sozial- und Gesundheitswesen durchzuführen. Es wird kontinuierlich weitergearbeitet, um diese Limitation zu reduzieren.

Da die Zugänglichkeit für die Anwender*innen bei den Instrumenten ebenfalls geprüft wurde, konnten diejenigen Instrumente nicht in den Review-Prozess einbezogen werden, die auch nach Kontaktaufnahme mit den Autor*innen nicht zur Sichtung zur Verfügung standen.

Der Ressourcenaufwand für eine kontinuierliche Aktualisierung der Übersichtsmatrix ist eine Herausforderung in personeller Hinsicht, sowohl für die wiederkehrende Recherche und den Review-Prozess als auch für die Pflege der Unterlagen. Die Arbeit bleibt eine Momentaufnahme, die kontinuierlich erweitert wird für die Arbeitsmediziner*innen, Arbeitsschutzakteure und Anwender*innen.

## Fazit für die Praxis

- Für die GBU Psyche, ein wichtiges Instrument zur Gestaltung der Arbeitsbedingungen in Einrichtungen, gibt es ein unübersichtliches Angebot an Analyseverfahren.
- Ein Hindernis bei der Auswahl des Verfahrens ist die Unsicherheit bei der Messbarkeit der psychischen Belastung.
- Für Anwender*innen wird eine reduzierte Auswahl an geeigneten Instrumenten für das Sozial- und Gesundheitswesen vorgestellt.
- Die Kriterien zur Beurteilung der Instrumente werden transparent beschrieben.

## Footnotes

1 Weitere Informationen zu den Steckbriefen finden sich unter dem folgenden Link: https://www.bgw-online.de/bgw-online-de/themen/sicher-mit-system/gefaehrdungsbeurteilung/instrumente-verfahren-zur-gefaehrdungsbeurteilung-psychischer-22292.

2 z. B. http://www.bgw-online.de.
